# Case report: A pediatric case of MPO-ANCA-associated granulomatosis with polyangiitis superimposed on post-streptococcal acute glomerulonephritis

**DOI:** 10.3389/fped.2023.1148132

**Published:** 2023-07-10

**Authors:** Qianhuining Kuang, Xu He, Lili Jia, Zhiqiang Zhang, Chunhong Gui, Chunlin Gao, Zhengkun Xia

**Affiliations:** Department of Pediatrics, Jinling Hospital, Nanjing, China

**Keywords:** ANCA-associated vasculitides, post-streptococcal acute glomerulonephritis, granulomatosis with polyangiitis, MPO-ANCA, immunosuppressive therapy, immunosuppressive therapy

## Abstract

An eight-year-old girl was admitted with vomiting, gross hematuria, and progressive renal dysfunction. A renal biopsy revealed endocapillary proliferative glomerulopathy and crescent formation. Immunofluorescence staining revealed diffuse granular deposits of IgG and C3. Post-streptococcal acute glomerulonephritis (PSAGN) was suspected, based on the elevated anti-streptolysin O levels, decreased serum C3 concentrations, and histologic findings. The myeloperoxidase anti-neutrophil cytoplasmic antibody (MPO-ANCA) test was positive, and the young patient gradually developed palisaded neutrophilic and granulomatous dermatitis (PNGD), orbital and paranasal sinus granulomatous neoplasms, along with intermittent nose, head, and orbital pain. Finally, she was diagnosed with the rare MPO-ANCA-associated granulomatosis with polyangiitis (GPA) superimposed on PSAGN. The patient was treated with aggressive renal replacement therapy, methylprednisolone pulse therapy, and intravenous pulse cyclophosphamide; her renal function normalized, and her pain symptoms improved.

## Introduction

Antineutrophil cytoplasmic antibody-associated vasculitides (AAVs) are a group of life-threatening primary systemic necrotizing small-vessel vasculitides characterized by the presence of antineutrophil cytoplasmic antibodies (ANCA), including granulomatosis with polyangiitis (GPA), microscopic polyangiitis (MPA), and eosinophilic granulomatosis with polyangiitis (EGPA), classified by clinical features. GPA and MPA frequently affect older adults, while EGPA tends to affect young adults. Approximately 60% of AAV cases involve the kidney; this condition is called ANCA-associated glomerulonephritis (AAGN), which can be diagnosed by a renal biopsy and typically manifests as necrotizing and crescentic glomerulonephritis with little to no immunoglobulin (Ig) or complement deposition. Cases progress to end-stage renal disease (ESRD) in 20%–25% of patients, and the renal prognosis of AAV is usually very poor.

Post-streptococcal acute glomerulonephritis (PSAGN) is an infection-related glomerulonephritis (IRGN) caused by group A β-hemolytic streptococci and characterized by endocapillary hypercellularity with immunoglobulin G (IgG) and complement (C3) deposition. Renal damage in PSAGN is caused by the activation of the classical and alternative complement pathways secondary to streptococcal-initiated humoral immunity. In the early stages of PSAGN, serum C3 levels are low and gradually return to the normal range within two months. PSAGN usually has a favorable renal prognosis and requires only suggestive therapy.

In this report, we describe a pediatric case of PSAGN, as suggested by laboratory test results and a renal biopsy, with progressive renal dysfunction that was alleviated by hemodialysis and immunosuppressive treatment. At baseline, MPO-ANCA was detected; during the course of the disease, granulomatous dermatitis and orbital and paranasal sinus granulomas gradually appeared. One case of AAV superimposed on a PSAGN presented with more severe renal dysfunction; however, the exact etiology of this condition remains unknown.

## Case description

An eight-year-old Chinese girl was admitted with complaints of vomiting, oliguria (150–300 ml/day), and gross hematuria for two days. She had no symptoms of fever, skin infection, or joint pain. Her blood pressure was elevated (122/80 mmHg). Serum laboratory tests revealed mild anemia (hemoglobin 96 g/L), severe acute kidney injury (blood urea nitrogen 35.1 mmol/L, creatinine 564.9 µmol/L, uric acid 610 µmol/L), and abnormal urine tests (185.6 RBCs/HPF, urine protein 0.63 g/24 h). Serum C3 concentration was 0.051 g/L (normal range: 0.9–1.8 g/L), anti-streptolysin O(ASO) was 682 IU/ml, and erythrocyte sedimentation rate (ESR, 54 mm/h) was elevated. Anti-nuclear antibody (1:100), anti-histone antibody+, MPO-ANCA 38.88R U/ml (by ELISA, normal range:<20 RU/ml), p-ANCA (+). Tests for other laboratory indicators, including procalcitonin, C-reactive protein (CRP), tumor markers, anti-cardiolipin antibody, and anti-glomerular basement antibody were negative. CT scans of the chest and abdomen were normal. Renal ultrasonography showed that the size of the right kidney was 115 × 47 mm^2^, the left kidney was 114 × 46 mm^2^, and the echo of the renal cortex was enhanced.

**Figure 1:**
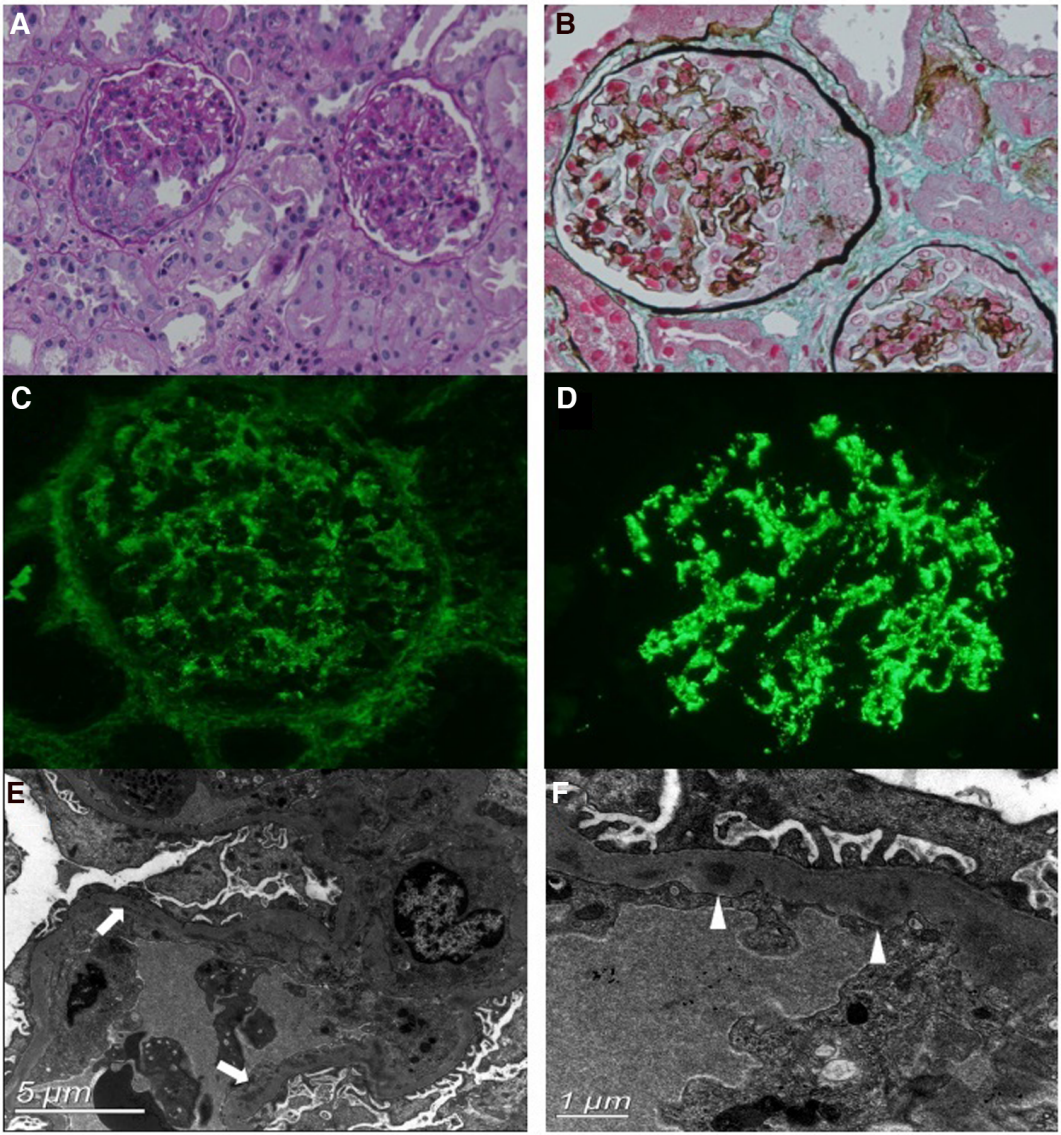
Renal biopsy findings. (**A,B**) Light microscopy (PAS and Masson's stain, ×200 magnification) shows the cellular crescent formation and diffuse inflammatory cell infiltration. (**C,D**) Immunofluorescence staining shows diffuse, granular deposits of IgG (**C**) and C3 (**D**) along the mesangial region and glomerular capillary walls. (**E,F**) Electron microscopy reveals electron-dense deposits in the segmental subendothelial region (arrows) and glomerular basement membrane (GBM) (triangles).

A renal biopsy was performed. Light microscopy revealed that among the 22 glomeruli, eight had cellular crescent formation and one had segmental sclerosis (Figures 1A,B). Furthermore, diffuse inflammatory cell infiltration, predominantly neutrophils and macrophages, was observed in the glomerular capillary lumen. The capillary wall appeared to be thickened. The tubulointerstitium displayed moderate acute injury, and the focal brush border of the tubular epithelium was detached. Immunofluorescence staining revealed diffuse granular deposits of IgG and C3 (IgG +, C3 +++) along the mesangial region and glomerular capillary walls (Figures 1C,D). Electron microscopy revealed electron-dense deposits in the mesangium and glomerular basement membrane (GBM) and intermittently in the sub-endothelial region (Figures 1E,F). No sub-epithelial electron-dense deposits were observed. In short, the renal pathology revealed endocapillary proliferative glomerulopathy. Complement component analysis and whole-exon sequence gene detection were normal.

**Figure 2:**
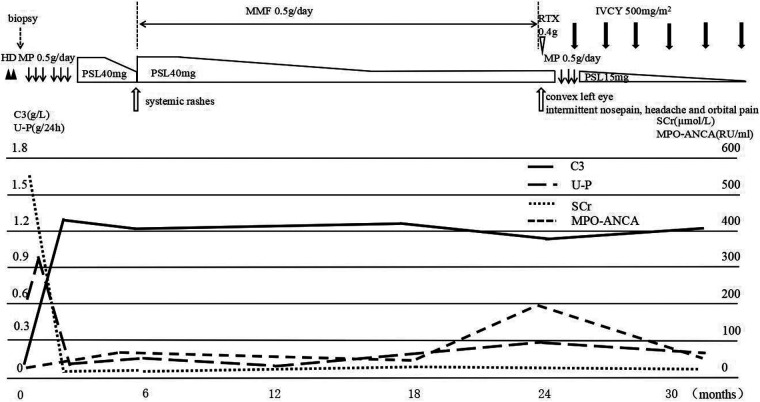
The clinical course of the patient. After hemodialysis and two pulses of methylprednisolone (500 mg/day), followed by 40 mg oral prednisone per day, serum C3 and SCr levels returned to normal, while MPO-ANCA was still positive. Subsequently, the patient developed systemic rashes; prednisone was increased to 40 mg/day, and mycophenolate was prescribed. Two years later, the girl presented with orbital and paranasal sinus abnormalities, the MPO-ANCA level was highly elevated, treatment with one dose of rituximab was unsuccessful, and another pulse of methylprednisolone combined with monthly IVCY brought the disease under control. MP, methylprednisolone pulse; PSL, prednisolone; IVCY, intravenous cyclophosphamide; U-P, urine protein.

Based on the clinical and histologic findings, the patient was diagnosed with PSAGN. However, it seemed to be an atypical case of PSAGN. Given the young patient's rapidly progressive clinical course and extensive crescent formation confirmed by renal biopsy, she was administered two pulses of methylprednisolone (500 mg/day, 20 mg/kg), followed by 40 mg (1.6 mg/kg) of oral prednisone per day, and hemodialysis was performed twice. After 20 days of treatment, serum creatinine levels returned to 29.9 µmol/L, hemoglobin increased to 114 g/L, and urine protein also decreased to 0.1 g/24 h. The serum C3 level was 1.3 g/L. Blood pressure (98/62 mmHg) and increased kidney size (96 × 37 mm^2^, 100 × 41 mm^2^) also went back to normal. Considering that the laboratory indicators returned to normal, other immunosuppressive agents were not added temporarily, and oral prednisone was tapered (Figure 2).

**Figure 3:**
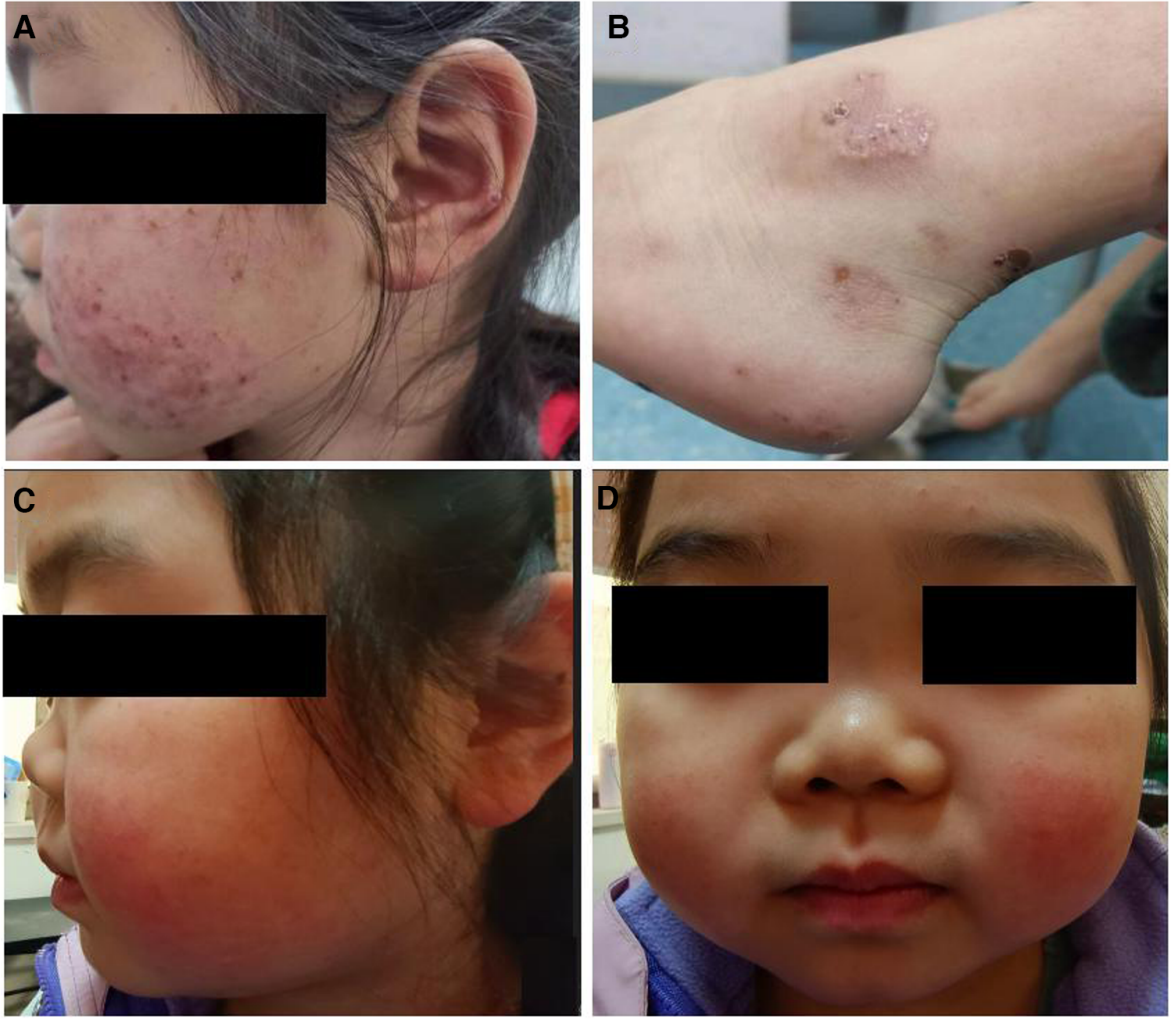
Clinical characteristics of the patient. (**A,B**) Systemic red rash. (**C,D**) Cartilage involvement, showing saddle nose deformity.

Approximately five months later, the patient developed recurrent systemic red rashes (Figures 3A,B). Laboratory tests revealed normal urine tests and renal function, p-ANCA+, MPO-ANCA 71.58 RU/ml, and a skin biopsy showed palisaded neutrophilic and granulomatous dermatitis (PNGD). Therefore, prednisone was added at 40 mg/day, and mycophenolate mofetil (20 mg/kg) was prescribed to control ANCA activation. The rash gradually resolved (Figure 2).

One and a half years later, the patient presented with a convex left eye and intermittent nose, head, and orbital pain with normal hearing. The bridge of the nose was low and flat, resembling a saddle nose (Figures 3C,D). Imaging revealed orbital pseudotumors and bilateral sinusitis. The patient underwent eye surgery, and the pathology of the left ocular mass indicated chronic inflammation with granulomas and necrosis. We simultaneously prescribed prednisone (15 mg/day), a single dose of rituximab (0.4 g, 375 mg/m^2^), and suspended mycophenolate mofetil. However, the patient's symptoms of nose, head, and orbital pain did not improve, ESR and CRP were elevated (ESR 38 mm/h, CRP 19.6 mg/L), p-ANCA was still positive, and MPO-ANCA was 193 RU/ml. A sinus biopsy revealed numerous granulomatous neoplasms in both maxillary sinuses. Consequently, the patient was administered one pulse of methylprednisolone (500 mg/day) for three days, followed by oral prednisone at 15 mg/day. Pulsed cyclophosphamide was then administered at 500 mg/m^2^ per dose for six doses every month. During treatment, the patient's urine and renal function remained normal, and there were no apparent lung lesions. The pain in the nose, eyes, and head gradually subsided.

## Discussion and conclusions

ANCA is usually thought to be the pathogen in AAV, which is an autoantibody that is directly against the cytoplasm of neutrophils and monocytes and includes mainly cytoplasmic (C-ANCA) and perinuclear (P-ANCA) forms. ANCA is specific to the diagnosis of GPA and MPA. C-ANCA occurs in 95% of new-onset GPA cases, P-ANCA in 80% of new-onset MPA cases, and 40% of new-onset EGPA cases. The prevalence of AAV is approximately 200–400 per million people, and it is rare in children (1). In children, AAV has a higher female preponderance. The peak incidence of AAV occurs in the second decade of life; the median age at diagnosis is 12–14 years; and GPA is more common than MPA or EGPA (2). Clinically, the prognosis of AAV is poor, and immunosuppressive therapy is warranted.

In our case, the clinical course of rapidly progressive abnormal renal function, positive MPO-ANCA at disease onset, and massive crescent formation proven by renal biopsy suggested the possibility of an AAV diagnosis. Subsequently, the girl developed PNGD, orbital and paranasal sinus granulomatous neoplasms, and a saddle nose deformity, accompanied by persistently positive MPO and P-ANCA according to the American College of Rheumatology/European Alliance of Associations (ACR/EULAR) (3) classification criteria, and was diagnosed with GPA. GPA was first described in 1937. The main clinical manifestations of GPA are granulomatous inflammation (orbital pseudotumor, chronic sinusitis, Eustachian tube dysfunction, etc.) and small- or medium-vessel vasculitis (pulmonary hemorrhage, glomerulonephritis, skin purpura, etc.). Upper airway abnormalities, manifesting as chronic rhinitis and serous otitis, may be the earliest presenting features and are present in more than 90% of cases, while abnormalities in the kidneys and lungs are present in 80% and 85% of cases, respectively (4). Historically, GPA has been diagnosed based on the presence of a histopathologic triad including necrotizing angiitis, granulomatous inflammation, and necrotizing crescentic glomerulonephritis (5). The incidence of GPA in the Asian population ranges from 0.37–2.1 per million people per year, and its prevalence in China is approximately 0.194 per million people. Most GPA patients in India and Korea are PR3 positive, while 60% of the GPA patients in China are MPO-ANCA positive (6). In addition, it has been confirmed that the ANCA serotype is more predictive of disease regression and clinical outcomes (7–9). A cohort analysis enrolled 365 patients diagnosed with AAV, 44 (12%) with MPA, and 321 (88%) with GPA. Among the 321 patients with GPA, 273 (85%) had PR3-ANCA, 33 (10%) had MPO-ANCA, and 15 (5%) remained ANCA negative. Compared with MPO-ANCA-positive MPA patients, MPO-ANCA-positive GPA patients were younger at diagnosis, and MPO-ANCA-positive GPA was predominantly female (10). Another case-control study showed that patients with MPO-ANCA-positive GPA were less likely to have severe disease (11) and had lower mortality and higher relapse rates (12). Therefore, it seems that some differences exist between MPO-ANCA-positive and PR3-ANCA-positive patients with GPA in terms of clinical manifestations, they may be different diseases (13).

Finally, the patient was diagnosed with an MPO-ANCA-associated GPA. Although recurrent episodes occurred, no fatal alveolar hemorrhage developed, and renal function quickly returned to normal. These clinical features are consistent with the above observations.

The pathogenesis of AAV is still poorly understood. Two familiar hypotheses are the complementary peptide model and the molecular mimicry model. However, evidence has shown that other immune cell mediators, such as CD4+ T cells, may be involved in AAV onset. Complement activation has been associated with the pathogenesis and progression of AAV. Some studies have reported immunocomplex (IC) formation during the early phases of AAV (14, 15). IC deposition is common in patients with AAGN, and C3 deposition is found in 30%–40% of patients with AAGN and is also an independent risk factor for AAV prognosis (16, 17). The renal biopsy of this patient revealed diffuse granular deposits of IgG and C3 (IgG +, C3 +++), which were incompatible with pauci-immune glomerulonephritis and showed more severe renal involvement in the early stage of the disease course, requiring renal replacement therapy.

ANCA can be caused by environmental exposures (silica), drug use (hydralazine, propylthiouracil, penicillamine, etc.), and disease (chronic inflammatory diseases, neoplasms, and infections). ANCA can also be caused by infection, and the possible mechanisms linking infection and ANCA include the production of neutrophil extracellular traps (NETs) and the ligation of toll-like receptors (TLRs) (18). Infection is associated with the morbidity of various glomerular diseases; the recently proposed concept of streptococcal infection-related nephritis (SIRN) includes IgAN, AAV, PSAGN, and so on. Streptococcal infection and ANCA act synergistically or coincidentally; however, the exact mechanism underlying the streptococcal infection associated with AAV remains unclear. In general, PSAGN patients had a good prognosis in the absence of immunosuppressive therapy. Crescent formation is not rare in PSAGN and are a predictor of poor long-term prognosis (19). In New Zealand children, 41% (11 of 27) of patients with crescentic PSAGN had higher serum creatinine levels, requiring acute dialysis (20). In addition, PSAGN can be associated with many other diseases, such as atypical hemolytic uremic syndrome (aHUS) (21), arthritis (19), IgAN (22), membranous nephropathy (23), and Alport syndrome (22). The co-occurrence of PSAGN and AAV is relatively rare (24, 25). The critical task is to decide whether additional treatment of the ANCA-associated disease is needed or only treatment of the infection. Ardiles et al. (26) tested serum IgG-ANCA levels in 210 patients with PSAGN and 14 patients with streptococcal impetigo without glomerular disease. In the PSAGN group, ANCA was detected in 9% of the patients. However, none of the subjects with streptococcal impetigo tested positive in this study. ANCA was associated with higher serum creatinine levels and more crescent formation, suggesting that ANCA may play a pathogenic role in kidney disease. Therefore, we prescribed glucocorticoids, and the renal function and urine abnormalities recovered quickly. When orbital and paranasal sinus granulomatous neoplasms developed, the combination of prednisone and pulsed cyclophosphamide controlled the disease.

In conclusion, we reported a rare case of MPO-ANCA-associated GPA superimposed on post-streptococcal acute glomerulonephritis, in which the prognosis was relatively good after active hemodialysis and immunosuppressive treatment. In cases of PSAGN with ANCA, although the initial ANCA titer is not very high, we recommend referring to the treatment principle of AAV, and aggressive and long-term maintenance of immunosuppressive therapies is required. A closer follow-up is warranted to focus on extrarenal manifestations and ANCA levels. Given the small number of cases of PSAGN with AAV, the pathophysiologic features and long-term renal prognosis remain unclear.

## Data Availability

The original contributions presented in the study are included in the article, further inquiries can be directed to the corresponding authors.
